# Efficacy of gel immersion endoscopic submucosal dissection for colorectal lesions

**DOI:** 10.1055/a-2865-3568

**Published:** 2026-06-02

**Authors:** Ken Inoue, Naohisa Yoshida, Reo Kobayashi, Takeshi Yasuda, Naoto Iwai, Osamu Dohi, Kazuhiko Uchiyama, Yuri Tomita, Ryohei Hirose, Hardesh Dhillon, Tomohisa Takagi

**Affiliations:** 1 Molecular Gastroenterology and HepatologyGraduate School of Medical Science, Kyoto Prefectural University of MedicineKyotoJapan; 2 Department of GastroenterologyKyoto Hakuaikai HospitalKyotoJapan; 3 Department of Infectious DiseaseKyoto Perfectural University of MedicineKyotoJapan; 4 Department of Gastroenterology 569275 Barwon HealthVictoriaAustralia

**Keywords:** Endoscopy Lower GI Tract, Colorectal cancer, Endoscopic resection (polypectomy, ESD, EMRc, ...), Quality and logistical aspects, Performance and complications

## Abstract

**Background and study aims:**

Endoscopic submucosal dissection (ESD) for colorectal lesions has become widely accepted but remains technically demanding, with perforation risk related to luminal distension and suboptimal visualization. Gel immersion ESD (GI-ESD) may offer mechanistic advantages over conventional CO ^2^ -insufflation ESD (C-ESD), including reduced colonic distension, improved visibility/depth perception, buoyancy-assisted dissection stability, and heat-sink protection. We hypothesized that these features would improve short-term safety and procedure stability.

**Patients and methods:**

In this single-center retrospective study, 426 consecutive colorectal ESD cases (from January 2022 to December 2024) were analyzed. C-ESD was performed from January 2022 to March 2024; GI-ESD using Viscoclear (Otsuka Pharmaceutical Factory, Tokushima, Japan) from April to December 2024. After excluding non-epithelial lesions, 392 patients were included, including interrupted procedures. The primary outcome was adverse events (perforation, delayed bleeding). Secondary outcomes were en bloc and R0 resection rates, procedure time, and postoperative inflammatory response.

**Results:**

Of 392 patients, 293 underwent C-ESD and 99 underwent GI-ESD were analyzed. En-bloc and R0 resection rates were comparable between GI-ESD and C-ESD (97.0% vs. 99.9%, *P* = 0.16, 90.6% vs. 91.4%, *P* = 0.35). The perforation rate was significantly lower in GI-ESD (0% vs. 4.1%, *P* = 0.04). Postoperative fever (≥ 37.6°C; 4.2% vs. 12.7%, *P* = 0.02) and median CRP elevation (0.33 mg/dl vs. 0.42 mg/dl, *P* < 0.01) were also significantly reduced in GI-ESD; however, incidence of post-endoscopic submucosal dissection electrocoagulation syndrome (PECS) was not significantly different between groups.

**Conclusions:**

GI-ESD achieved curative outcomes comparable to C-ESD while reducing perforation risk; PECS remained comparable between groups.

## Introduction

Endoscopic submucosal dissection (ESD) to treat colorectal cancer has an excellent curative resection rate 1 . Despite its proven efﬁcacy, widespread adoption of colorectal ESD remains limited due to the higher risk of severe adverse events (AEs), such as perforation, compared with endoscopic mucosal resection (EMR) 2 .

In recent years, underwater and water pressure method ESD (WPM-ESD) have been investigated as an alternative method to overcome technical challenges of colorectal ESD 3 4 5 . By using saline immersion, these techniques improve endoscopic visualization and submucosal access through mucosal buoyancy and reduced intraluminal pressure. However, their clinical utility remains limited by intermittently obscured visual fields caused by residual liquid and air bubbles.

In 2021, a novel gel formulation for endoscopic procedures (Viscoclear; Otsuka Pharmaceuticals Factory, Tokushima, Japan) was launched in Japan. This gelatinous solution, composed of xanthan gum, locust bean gum, and glycerin, does not mix with intestinal fluids or blood, thereby maintaining a clear endoscopic view throughout the procedure 6 7 .

Clinical efficacy of this gel in identifying active sources of gastrointestinal bleeding has been demonstrated in numerous studies 8 9 10 . Furthermore, low-pressure endoscopy using the gel immersion method has been shown to facilitate endoscopic reduction of a Morgagni hernia 11 . Minimization of halation, bubbles, and intestinal fluid inflow contributes to excellent visualization, with diagnostic accuracy comparable to that achieved with CO _2_ insufflation and underwater conditions 12 .

**Fig. 1:**
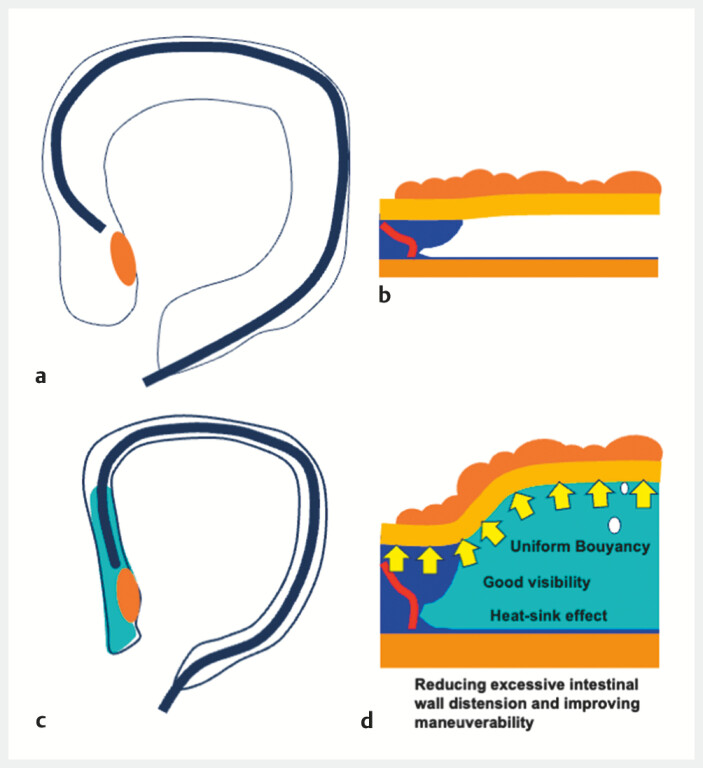
Study flow.

In endoscopic resection, the gel immersion technique for EMR of gastric, duodenal and colorectal tumors enables continuous lumen filling that maintains adequate bowel distention, providing a stable visual field that enhances procedure efficiency and safety 13 14 15 16 17 . Building on these advantages, gel immersion has also been applied to ESD as an effective alternative to conventional ESD (C-ESD) for esophageal and duodenal tumors 18 19 20 . Gel immersion during ESD offers improved mucosal buoyancy, facilitating submucosal access, and maintains a low-pressure environment without air or CO _2_ insufflation, thereby improving procedure stability and maneuverability compared with C-ESD 18 19 . Although a few reports have described feasibility of GI-ESD for colorectal tumors, 21 22 23 24 25 larger-scale evidence remains limited. Use of gel during colorectal GI-ESD is intended to provide several mechanistic advantages over C-ESD: reduced colonic distension with improved endoscopic maneuverability, enhanced visibility/depth perception, buoyancy-assisted stabilization of trimming and submucosal dissection, and attenuation of thermal injury via a sink effect 18 19 20 26 . Based on these features, we hypothesized that GI-ESD would yield improved short-term safety and procedure stability compared with C-ESD. Therefore, the aim of this study was to test whether the hypothesized mechanistic advantages of GI-ESD translate into improved short-term outcomes, particularly safety and procedure stability, compared with conventional C-ESD ( Fig. 1 ).

## Patients and methods

**Fig. 2:**
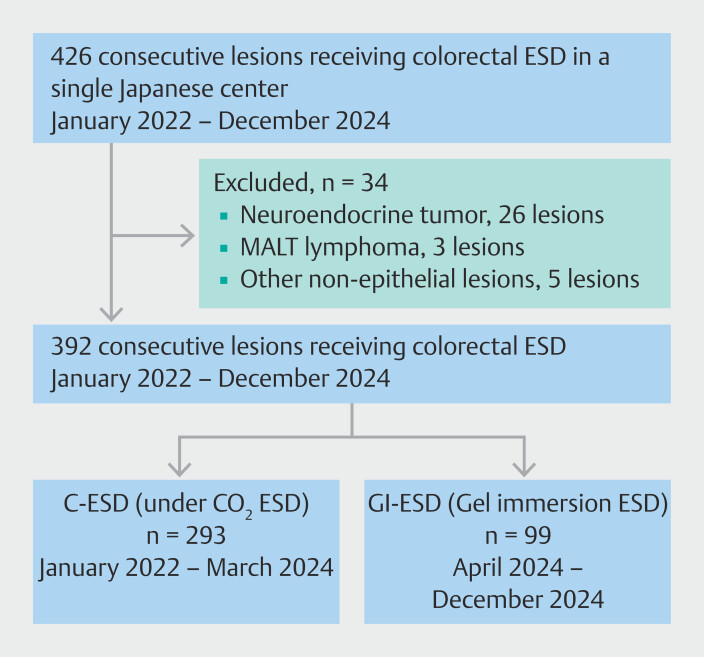
Conceptual comparison between conventional ESD and gel immersion ESD in colorectal lesions. **a** C-ESD: Excessive luminal insufflation causes marked colonic wall distension. **b** C-ESD during dissection under gas insufflation. **c** GI-ESD: gel immersion suppresses excessive colonic distension and maintains a more stable operative field. **d** Proposed advantages of GI-ESD: reduced excessive colonic wall distension (improving endoscopic maneuverability), improved visibility and depth perception in a stable gel medium, uniform buoyancy facilitating controlled trimming and submucosal dissection, and a heat-sink effect that may reduce local thermal injury to the muscularis propria. C-ESD, conventional endoscopic submucosal dissection; GI-ESD, gel immersion endoscopic submucosal dissection.

This was a single-center retrospective study. A total of 426 consecutive patients who underwent colorectal ESD at Kyoto Prefectural University of Medicine between January 2022 and December 2024 were enrolled ( Fig. 2 ). Indications for ESD followed Japanese guidelines and included lesions ≥ 20 mm in size that were deemed unsuitable for en bloc EMR. All lesions were examined with magnified endoscopy to determine suitability for endoscopic resection 27 . Patients with non-epithelial colorectal tumors were excluded. Written informed consent for colorectal ESD was obtained from all patients. The study was approved by the Institutional Review Board of the Kyoto Prefectural University of Medicine (ERB-C-1600–3, Acceptance date: June 5, 2025) in accordance with the ethical standards outlined by the latest amendment of the 1964 Helsinki Declaration.

All colorectal ESD procedures were carried out with an EC-L600ZP7, EC-760ZP-V/M (Fujifilm Co., Tokyo, Japan) or PCFH290ZI (Olympus Co., Tokyo, Japan) endoscope. A transparent distal attachment cap, (Elastic touch, Top Corporation, Tokyo, Japan) or a small-caliber-tip transparent hood (Fujifilm Co., Tokyo, Japan) was attached to the endoscope tip. ESD procedures were conducted under sedation with midazolam (Dormicum; Astellas Pharma, Tokyo, Japan) or propofol (Maruishi Pharmaceutical Co., Ltd., Osaka, Japan) with pentazocine (Pentazin; Sankyo Pharmaceuticals, Tokyo, Japan) or pethidine (Pethidine; Takeda Pharmaceutical Co., Ltd., Osaka, Japan).

Sodium hyaluronate (MucoUp; Boston Scientific, Tokyo, Japan) or sodium alginate (Lifthal K; Kaigen Co., Ltd., Osaka, Japan) mixed with a small amount of indigo carmine was used as the injection solution to achieve a sustained submucosal elevation. All ESDs were performed using a 3.5-mm Clutch Cutter (Fujifilm Co., Tokyo, Japan). Use of a traction device was at the discretion of the endoscopist; when used, either the SO clip (Zeon Medical Co., Ltd., Tokyo, Japan) or SureClip Traction Band (Micro-Tech, Nanjing, China) was employed.

With respect to bowel preparation, patients consumed a low-residue diet and 10 mL of sodium picosulfate the day before the examination. On the morning of the procedure, all patients received 1.0 L of a highly concentrated polyethylene glycol solution with ascorbic acid (MOVIPREP; EA Pharma Co., LTD, Tokyo, Japan).

### GI-ESD procedure

Gel immersion ESD. A superficially elevated lesion with a nodule in the sigmoid colon. Mucosal incision was performed under gel immersion. Submucosal dissection was performed using the pocket-creation method. ESD was performed without complications in 93 minutes. En-bloc resection was achieved.Video 1

All GI-ESDs were performed after April 2024, whereas C-ESDs were performed between January 2022 to March 2024 ( Video 1 ). In this study, a procedure was classified as gel immersion ESD (GI-ESD) when part or all of the mucosal incision and submucosal dissection was performed under the clear gel solution (VISCOCLEAR; Otsuka Pharmaceuticals Factory, Tokushima, Japan), whereas conventional ESD (C-ESD) was defined as procedures in which all steps were performed without the gel solution. CO _2_ was used for insufflation instead of air for all ESD procedures.

After deflating the colorectal lumen, the gel solution was injected via the accessory channel of the endoscope using a foot pump system. When bubbles or bleeding appeared during mucosal incision or submucosal dissection, additional gel was injected to maintain visibility and facilitate identification of bleeding points.

A high-frequency electrical generator (VIO3; Erbe Elektromedizin) was used for all ESDs with the following settings: Endocut I mode, effect 1, duration 4, interval 1 for mucosal incision and submucosal dissection; Forced Coagulation mode, effect 3.0 for submucosal dissection with minor vessels; and soft coagulation mode, effect 3.0-5.0 or Forced Coagulation mode, effect 1.0 for precoagulation of vessels and hemostasis..

For cases at high risk of delayed bleeding or perforation, post-ESD defect closure was performed using various clipping devices and techniques at the discretion of the individual endoscopist.

All colorectal ESDs were performed by one of two expert endoscopists (N.Y. and K.I.) or by one of 15 less-experienced endoscopists. The expert endoscopists had performed ≥ 300 prior colorectal ESD procedures using the Clutch Cutter, whereas the less-experienced endoscopists had performed < 20 at the beginning of the study period. Before performing ESDs under the direct supervision of an expert (N.Y. or K.I.), using the Clutch Cutter, all less-experienced endoscopists observed expert-performed procedures for at least 1 month. Supervisors took over the procedure in cases where the estimated procedure time for mucosal incision and submucosal dissection was expected to exceed 60 minutes or when an AE occurred. The less-experienced endoscopist was allowed to resume the procedure if the supervisor had successfully managed the issue (e. g. bleeding or perforation).

AEs were defined as follows 28 . Intraprocedure perforation was defined as a visible defect in the muscularis propria with exposure of fatty tissue or adjacent organs, confirmed during the ESD procedure. Delayed bleeding was defined as post-procedure bleeding requiring endoscopic hemostasis or a decrease in hemoglobin by ≥ 2 g/dL within 30 days post ESD. Delayed perforation was defined as presence of free air on computed tomography within 14 days after ESD, which was not attributable to intraprocedure perforation. Post-ESD coagulation syndrome (PECS) was defined as localized abdominal tenderness with a fever (≥ 37.6°C) or inﬂammatory response (leukocytosis [≥ 10000 cells/mL] or raised C-reactive protein [CRP] [≥ 0.5 mg/dL]) without deﬁnite evidence of perforation (ie, no extraluminal free air) occurring ≥ 6 hours after colorectal ESD 29 .

Morphologically flat polyps were classified according to the Paris classification 30 . Resected specimens were fixed in 10% formalin before histological evaluation. Histopathological diagnosis was made by three clinical pathologists according to the World Health Organization (WHO) classification 31 , with intramucosal cancer reclassified as high-grade dysplasia (HGD). Serrated lesions, including sessile serrated lesions (SSLs) and SSL with dysplasia (SSLD), were also diagnosed in accordance with the WHO classification.

### Outcomes

The primary outcome of the study was the difference in AE rates between C-ESD and GI-ESD cases. Secondary outcomes included en bloc and R0 resection rates, procedure time, and post inflammatory response, as assessed by white blood cell (WBC) count and CRP level. Procedure time was defined as the interval from the start of local injection to completion of the procedure (completion of en bloc resection) and included the time required for gel injection.

### Statistical analysis

Comparisons between the two ESD groups were performed using the chi-squared test or Fisher’s exact test for categorical variables and the Mann–Whitney U tests or Student’s *t* -tests for continuous variables. Statistical analyses were performed using the Statistical Package for the Social Sciences software (SPSS version 25.0; IBM Corp., Armonk, New York, United States). Statistical significance was set at *P* < 0.05.

## Results

A total of 426 patients who underwent ESD for colorectal tumors were enrolled. After excluding 34 patients with non-epithelial lesions (neuroendocrine tumor, n = 26; MALT lymphoma, n = 3; degenerative colonic mucosa, n = 1; proctitis, n = 1; mucosal prolapse, n = 1; lymphoid hyperplasia, n = 1; condyloma acuminatum, n = 1), 392 patients were finally examined: 293 in the C-ESD group and 99 in the GI-ESD group ( Fig. 1 ).

**Fig. 3:**
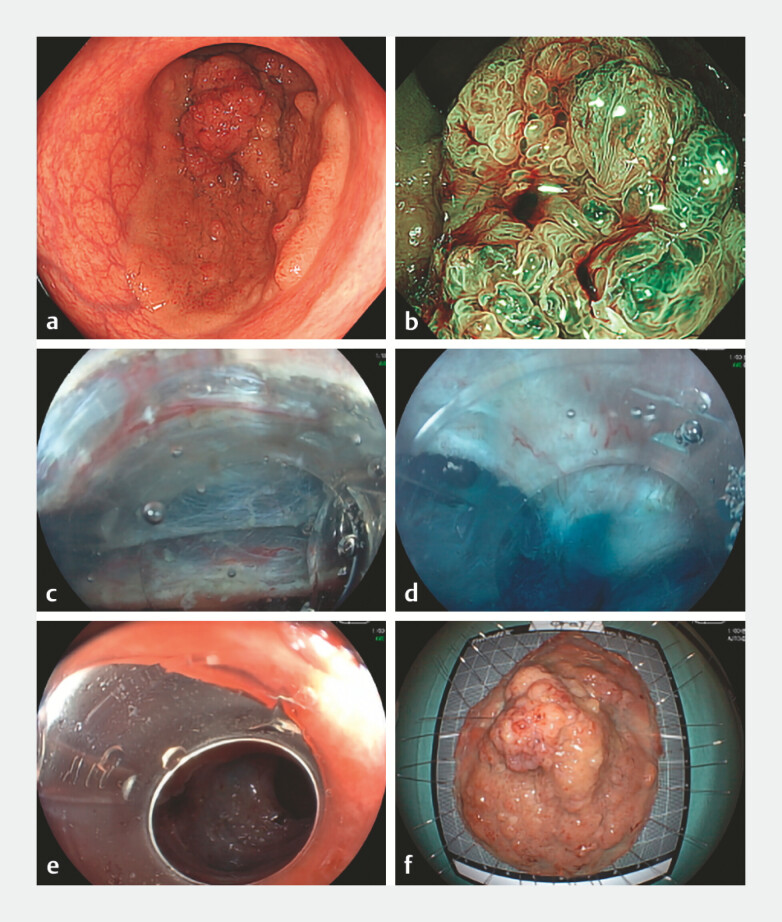
Case presentations. **a** A superficially elevated lesion with a nodule in the sigmoid colon. **b** NBI with magnification. **c** Mucosal incision was performed under gel immersion. **d** Submucosal dissection was performed using the pocket-creation method. **e** ESD was performed without complications in 93 minutes. **f** En-bloc resection was achieved (well-differentiated adenocarcinoma, 90 mm, pTis, Ly0, V0). ESD, endoscopic submucosal dissection.

Patient characteristics and ESD outcomes are summarized in Table 1 , Table 2 , Table 3 , and Fig. 3 . There were no significant differences between groups in median age, sex ratio, tumor location, morphology, mean tumor size, expert-to-less-experienced operator rate, body temperature, or baseline WBC count and CRP level.

**Table:** **Table 1** Baseline characteristics of all patients.

	C-ESD (n = 293)	GI-ESD (n = 99)	*P* value
Age (years), median (range)	70 (31–91)	73 (42–92)	0.11
Sex, male/female	181/112	63/36	0.74
Tumor location, n (%)			0.22
Cecum	40 (13.7)	17 (17.2)	
Ascending colon	58 (19.8)	25 (25.2)	
Transverse colon	63 (21.5)	27 (27.2)	
Descending colon	21 (7.1)	6 (6.1)	
Sigmoid colon	36 (12.3)	9 (9.1)	
Rectum	75 (25.6)	15 (15.2)	
Morphology, n (%)			0.20
Non-polypoid	228 (77.8)	83 (83.8)	
Polypoid	65 (21.9)	16 (13.5)	
Tumor size, mean (± SD), mm	31.8 ± 18.3	29.6 ± 15.0	0.29
Major diameter of resected specimen, mean (± SD), mm	41.9 ± 18.2	39.3 ± 15.2	0.21
Minor diameter of resected specimen, mean (± SD), mm	34.5 ± 13.7	32.6 ± 13.6	0.25
Operator, n (%)			0.18
Experts	155 (52.9)	60 (60.6)	
Less experienced	138 (47.3)	39 (40.6)	
Body temperature, median (range), °C	36.7 (35.7–37.3)	36.7 (36.0–37.4)	0.56
WBC count, median (range), µL	6,000 (1,400–16,100)	6,000 (3,700–12,600)	0.46
CRP, median (range), mg/dL	0.06 (0.01–5.47)	0.08 (0.01–1.98)	0.43
C-ESD, conventional endoscopic submucosal dissection; CRP, C-reactive protein; GI-ESD, gel immersion endoscopic submucosal dissection; PECS, post-endoscopic submucosal dissection coagulation syndrome; WBC, white blood cell.			

**Table:** **Table 2** Technical outcomes of ESD (C-ESD vs. GI-ESD).

	C-ESD (n = 293)	GI-ESD (n = 99)	*P* value
Procedure time, median (range), min			
All cases	54 (7–293)	52 (21–145)	0.20
Experts	43 (7–287)	45 (21–145)	0.29
Less experienced	60 (16–293)	60 (25–113)	0.57
Dissection speed, median (range), mm ^2^ /min			
All cases	19.4 (2.4–71.6)	16.9 (1.8–67.2)	0.37
Experts	22.5 (2.4–65.4)	20.9 (1.8–67.2)	0.49
Less experienced	16.5 (4.7–71.6)	14.8 (3.6–43.1)	0.17
Traction device, n (%)	205 (69.9)	77 (77.9)	0.13
Severe fibrosis, n (%)	55 (18.5)	19 (16.7)	0.93
Closure of ESD defect, n (%)	244 (83.3)	89 (89.9)	0.11
En bloc resection, n (%)	290 (99.0)	96 (97.0)	0.16
Histopathological complete resection (R0), n (%)	267 (91.1)	87 (87.9)	0.35
Histology, n (%)			0.33
Serrated lesions	43 (14.7)	9 (9.4)	
Adenoma	206 (70.2)	76 (77.1)	
Adenocarcinoma (≥ T1)	44 (15.1)	14 (13.5)	
Adverse events			
All cases			
Perforation, n (%)	12 (4.1)	0 (0)	0.04
Intraoperative perforation, n (%)	5 (1.7)	0 (0)	0.20
Delayed perforation, n (%)	7 (2.4)	0 (0)	0.13
Delayed bleeding, n (%)	4 (1.4)	3 (3.1)	0.26
Experts			
Perforation, n (%)	12 (7.8)	0 (0)	0.03
Intraoperative perforation, n (%)	5 (3.2)	0 (0)	0.17
Delayed perforation, n (%)	7 (4.5)	0 (0)	0.10
Delayed bleeding, n (%)	0 (0)	1 (1.8)	0.10
Less experienced			
Perforation, n (%)	0 (0)	0 (0)	-
Intraoperative perforation, n (%)	0 (0)	0 (0)	-
Delayed perforation, n (%)	0 (0)	0 (0)	-
Delayed bleeding, n (%)	4 (2.9)	2 (5.1)	0.50
Interrupted procedures	1 (0.3)	3 (3.0)	0.02
C-ESD, conventional endoscopic submucosal dissection; GI-ESD, gel immersion endoscopic submucosal dissection.			

**Table:** **Table 3** Detailed inflammation after ESD (C-ESD vs. GI-ESD).

	C-ESD (n = 293)	GI-ESD (n = 99)	*P* value
Body temperature, median (range), °C	37.0 (36.0–38.7)	36.9 (36.2–37.8)	0.04
Body temperature, ≥37.6°C, n (%)	37 (12.7)	4 (4.2)	0.02
Localized abdominal pain, n (%)	44 (15.1)	6 (6.3)	0.03
WBC count (POD1), median (range), µL	7,500 (2,400–22,700)	7,400 (4,300–15,700)	0.50
WBC count (POD2), median (range), µL	6,650 (1,600–15,200)	6,600 (3,700–13,900)	0.55
WBC count increase, median (range), µL	1,500 (0–15.300)	1,300 (0–5,100)	0.02
CRP (POD1), median (range), mg/dL	0.42 (0.01–12.06)	0.33 (0.02–3.59)	<0.01
CRP (POD2), median (range), mg/dL	1.28 (0.01–36.87)	1.09 (0.04–15.43)	0.02
CRP increase, median (range), mg/dl	1.09 (0–36.67)	0.83 (0.03–15.31)	<0.01
PECS, n (%)	29 (10.4)	6 (6.3)	0.23
C-ESD, conventional endoscopic submucosal dissection; CRP, C-reactive protein; GI-ESD, Gel immersion endoscopic submucosal dissection; PECS, post-endoscopic submucosal dissection coagulation syndrome; WBC, white blood cell.			

Rates of traction device use, severe fibrosis rate, and ESD defect closure did not differ significantly between the two groups. En bloc and R0 resection rates were 99.0% vs. 97.0% and 91.1% vs. 87.9% in the C-ESD and GI-ESD groups, respectively ( *P* = 0.16 and *P* = 0.35). Pathological diagnoses were compared between groups. Median resection time and median dissection speed between the C-ESD and GI-ESD groups were not significantly different (54 vs. 52 min and 19.4 vs. 16.9 mm ^2^ /min for C-ESD and GI-ESD, respectively).

The perforation rate was significantly higher in the C-ESD group than in the GI-ESD group (4.1% vs. 0%, respectively, *P* = 0.04). There was no significant difference in the rate of delayed bleeding between the two groups. All perforation events occurred in the experienced C-ESD subgroup. For delayed bleeding, one event occurred in the experienced GI-ESD subgroup; among non-experienced operators, delayed bleeding occurred in four cases (2.9%) in the C-ESD group and two cases (5.1%) in the GI-ESD group. Four interrupted procedures were included in the cohort: one case in the C-ESD group (0.3%) and three cases in the GI-ESD group (3.0%) ( *P* = 0.02). All four interrupted cases were semipedunculated lesions more than 30 mm in size with severe submucosal fibrosis and muscularis propria traction.

Mean body temperature between the C-ESD group and in the GI-ESD group did not differ significantly; however, the proportion of patients with a fever ≥ 37.6°C was significantly higher in the C-ESD group than in the GI-ESD group (12.7% vs. 4.2%, *P* = 0.02). Similarly, localized abdominal pain post-ESD was more frequent in the C-ESD group (15.1% vs. 6.3%, *P* = 0.03).

No significant differences were observed in median WBC count on postoperative Day 1 or 2 or in WBC rise between groups. In contrast, median postoperative CRP values were significantly higher in the C-ESD group compared with GI-ESD; postoperative day 1 (0.42 [0.01–12.06] vs. 0.33 [0.02–3.59] mg/dL, *P* < 0.01), postoperative Day 2 (1.28 [0.01–36.87] vs. 1.09 [0.04–15.43] mg/dL, *P* = 0.02), and CRP increase (1.09 [0.00–36.67] vs. 0.83 [0.03–15.31] mg/dL, *P* < 0.01). However, incidence of patients with PECS did not differ significantly between the two groups (10.4% vs. 6.3%, *P* = 0.23).

**Fig. 4:**
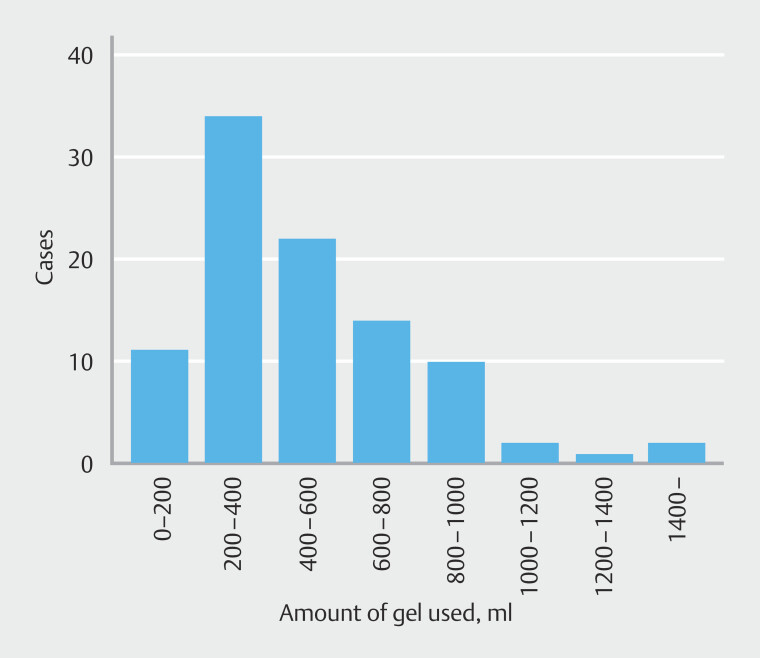
Amount of gel used during GI-ESD. GI-ESD, gel immersion endoscopic submucosal dissection.

For GI-ESD, mean gel volume used was 559 ± 387 mL per lesion (range: 120-2800 ml). Distribution of gel usage was as follows: < 200 mL in 11 cases (11.5%), 200 to 400 mL in 34 cases (35.4%), 400 to 600 mL in 22 cases (23.0%), 600 to 800 mL in 14 cases (14.6%), 800 to 1000 mL in 10 cases (10.4%), and > 1000 mL in five cases (5.1%) ( Fig. 4 ).

## Discussion

In this study, we compared efficacy and safety of colorectal GI-ESD compared with C-ESD. We evaluated perforation and delayed bleeding rates, en bloc and R0 resection rates, procedure time, and postoperative inflammatory response. GI-ESD demonstrated a significantly lower perforation rate and post-operative inflammatory response compared with C-ESD while achieving comparable en bloc and R0 resection rates and procedure time. To our knowledge, this is the first study to demonstrate a lower incidence of AEs with GI-ESD compared with C-ESD.

Previous reports have shown effectiveness of gel immersion in esophageal and duodenal ESD, but studies specifically addressing colorectal ESD remain limited 18 19 25 . Our results confirm that performing colorectal ESD under gel offers superior safety to CO₂ insufflation, with a significant reduction in perforation rate and lower postoperative inflammatory biomarker levels. The perforation rate in the C-ESD group (4.0%) was higher than that reported in some previous studies. Importantly, all perforation events in this group occurred during procedures performed by expert endoscopists, suggesting that technical immaturity was unlikely to be the main explanation. A more plausible explanation is a higher level of case difficulty (e.g., lesion-related complexity such as severe fibrosis and poor operability). Therefore, comparisons of perforation rates across studies should be interpreted with caution, given differences in case mix and reporting practices. We acknowledge that isolated elevations in WBC or CRP are not necessarily clinically meaningful. Therefore, postoperative inflammatory status should be interpreted primarily based on PECS (a composite of clinical symptoms/signs and inflammatory markers), whereas WBC/CRP trends should be regarded as supportive rather than definitive.

Gel immersion endoscopy is an innovative technique that replaces air or water with a viscous gel medium to improve visualization during gastrointestinal procedures 6 32 . Sato et al. described its utility in overcoming challenges with air insufflation, particularly by improving endoscopic visualization in anatomically difficult to access regions 33 . Previous studies have reported excellent outcomes with GI-ESD, including a 100% en bloc resection rate for esophageal and duodenal lesions 18 19 34 . Viscosity of the gel maintains a stable submucosal cushion, ensuring a clear visual field throughout dissection. GI-ESD also offers additional advantages, such as reduced muscle layer exposure (1.9% vs 16.7%) and lower post procedure inflammatory response 18 . The technique has demonstrated a favorable safety profile, with no gel-related AEs reported and effective hemostasis capabilities when bleeding occurs 18 19 .

GI-ESD appears particularly beneficial for lesions in challenging locations such as gravity-dependent esophageal lesions, achieving a median procedure time of 27 minutes and dissection speeds of 20 mm²/min 19 . Consistent with these reports, our study confirmed that colorectal GI-ESD offered superior safety outcomes compared with C-ESD, evidenced by lower perforation rates and post procedure inflammatory responses.

Improved safety and stability of GI-ESD over use of CO _2_ -insufflation can be attributed to several factors. First, avoiding gas insufflation minimizes colonic distension, improving endoscopic maneuverability. Second, Viscoclear gel enhances depth perception and image magnification by remaining immiscible with intestinal fluids while making objects appear approximately 1.3 times larger and 0.75 times closer 12 . Third, uniform buoyancy provided by the gel facilitates controlled trimming and submucosal dissection. Fourth, it exerts a heat-sink effect that limits local temperature elevation, preventing thermal injury to the muscularis propria. Finally, reduced colonic wall distention further reduces risk of perforation.

These advantages are comparable to those observed with underwater ESD and water pressure method ESD (WPM-ESD) for colorectal lesions 3 35 36 . However, saline immersion often results in a turbid visual field due to mixing with residual fluid and air bubbles. In contrast, gel immersion, by virtue of its viscosity, maintains a stable and clear field of view even in the presence of residual liquid or bleeding, facilitating precise identification of the dissection line and bleeding points. Moreover, this technique achieves these benefits with a modest gel volume. In our study, mean gel volume used was 559 ± 387 mL per lesion and no gel-related AEs were observed.

To evaluate potential impact of the learning curve, we performed an operator-stratified AE analysis (experienced vs non-experienced operators), similar to the subgroup analysis used for procedure time. All perforation events were observed in the experienced C-ESD subgroup. Delayed bleeding occurred in one experienced GI-ESD case, whereas among non-experienced operators, it occurred in four C-ESD cases (2.9%) and two GI-ESD cases (5.1%). Although these findings do not indicate that the observed safety differences were explained solely by operator maturation, the low event counts and retrospective design require cautious interpretation.

Importantly, all interrupted cases involved semipedunculated lesions with severe submucosal fibrosis and muscularis propria traction. This pattern indicates that endoscopic completion can be challenging in such lesions even when GI-ESD is used, similar to C-ESD. Therefore, these lesion characteristics may represent a technical boundary condition of GI-ESD and should be considered during case selection and procedure planning.

This study had several limitations. First, this was a single-center, retrospective, non-randomized, controlled study. Second, the GI-ESD group had a smaller sample size compared with the C-ESD group. Third, all procedures were performed using a single knife type (Clutch Cutter), which may limit generalizability. Fourth, in general, treatment procedures evolve over time naturally; thus, treatment outcomes, including curability and safety, can be better in the late phase of treatment periods.

Because GI-ESD was conducted in the late phase of treatment periods, technical maturity over time could lead to a better safety profile. Future multicenter, prospective, randomized studies with larger sample sizes are warranted to validate these findings and further assess the clinical utility of colorectal GI-ESD.

## Conclusions

In conclusion, colorectal GI-ESD achieves a high curative resection rate with a lower incidence of AEs, highlighting its potential as a safe and effective alternative to conventional ESD.

## Data availability statement

Patient data used to support the findings of this study are available from the corresponding author upon request. However, some data are restricted by the institutional review board of the Kyoto Prefectural University of Medicine.
